# Releasing the Spring: The Association Between Trunk-on-Pelvis Acceleration and Ball Velocity in Baseball Pitching

**DOI:** 10.3390/sports14100424

**Published:** 2026-10-01

**Authors:** Karl F. Orishimo, Zachary Amster, Ian J. Kremenic, Nicholas Serio, Malachy P. McHugh

**Affiliations:** 1 Nicholas Institute of Sports Medicine and Athletic Trauma Northwell Health, Lenox Hill Hospital, 210 E. 64th St., 5th Floor, New York, NY 10075, USA; 2 Athletes Warehouse, Pleasantville, NY 10570, USA

**Keywords:** velocity, kinematics, recoil, stretch-shortening cycle, throwing

## Abstract

The objective of this study was to compare the spring-like properties of pelvis and trunk rotation during baseball pitching in high school pitchers with varying ball velocities. It was hypothesized that high-velocity pitchers would be more effective at accelerating the trunk on the pelvis from foot contact to ball release. Three-dimensional kinematics were recorded in 35 high school pitchers (age 16.5 ± 1.1 year, height 1.83 ± 0.06 m, body mass 82.8 ± 9.9 kg, and pitch velocity 75.0 ± 5.6 mph). Pelvis and trunk angular velocities and accelerations, and trunk–pelvis separation, were calculated and averaged for 3 pitches. Additionally, average trunk-on-pelvis acceleration was calculated from foot contact to ball release. Pitchers were classified as high-, average-, or low-velocity, and single-factor ANOVA was used to compare metrics between groups. Peak pelvis and trunk velocities were higher for high- versus low-velocity pitchers (*p* = 0.001 and *p* = 0.008, respectively). Pelvis deceleration was greater in high-velocity pitchers (*p* = 0.024), and average trunk-on-pelvis acceleration was greater for high-velocity pitchers (*p* = 0.013). Acceleration of the trunk on the pelvis during the baseball pitch quantifies the spring-like properties derived from pelvis and trunk kinematic sequencing. High-velocity pitchers more effectively release the spring by decelerating the pelvis prior to ball release.

## 1. Introduction

Rotational athletic movements, such as baseball pitching, batting and the golf swing, are generally characterized by a proximal-to-distal sequence of segmental motion [1,2,3]. The sequential angular acceleration and deceleration of adjacent segments creates angular “separation”, which stores elastic energy and activates the stretch-shortening cycle in the muscles between the segments. The resulting release of rotational elastic energy during the ensuing muscle contraction, much like a torsional spring, accelerates the distal segment to a maximal angular velocity that is greater than its predecessor. This process continues between segments through the kinetic chain, building segmental velocities and resulting in the most distal segment (the hand in baseball pitching) achieving maximal velocity [2].

Previous biomechanical analyses of the pitching motion have shown that ball velocity can not only be improved by increasing upper-extremity joint velocities such as maximum elbow extension velocity or maximum shoulder internal rotation velocity [4,5] but also by increasing the maximum velocities of the pelvis and trunk [6,7,8]. Additionally, peak trunk velocity has been demonstrated to be a factor that differentiates higher-velocity pitchers from lower-velocity professional pitchers [9]. All other factors being equal, achieving greater peak trunk velocity implies greater release of rotational elastic energy between the pelvis and trunk and, therefore, greater trunk-on-pelvis acceleration. This suggests that higher-velocity pitchers may have better ability to release stored rotational energy during the pitching motion. Given the torsional spring analogy, the relative acceleration of the trunk to the pelvis may be a more appropriate measure to describe a pitcher’s ability to release rotational elastic energy than peak rotation velocity, and it may also differentiate performance.

To the authors’ knowledge, Fleisig et al. [10] is the only study to quantify the trunk-on-pelvis rotational acceleration during the pitching motion. This study found that peak trunk–pelvis separation and peak trunk-on-pelvis acceleration occurred around foot contact and attributed this instant of high stress to the simultaneous deceleration of the pelvis and acceleration of the trunk at this time point. However, separate trunk and pelvis accelerations and decelerations were not examined, and a full description of the pelvis and trunk interaction could not be provided. Additionally, this study did not attempt to correlate these metrics to performance measures, such as ball velocity.

In an analysis of golf swing biomechanics, McHugh et al. [1] examined separate trunk and pelvis accelerations and decelerations and their possible relationship with performance. This study found that proficient golfers had greater trunk and pelvis accelerations and decelerations than unskilled golfers during the downswing. These authors, however, did not include the relative acceleration of the trunk to the pelvis in their analysis, which may be a more sensitive differentiator of performance.

Having a complete picture of the interaction between the trunk and pelvis during the pitching motion, which includes both the individual segment, as well as the relative, kinematics (angular position, velocity and acceleration), can give valuable insight into the ability of a pitcher to release stored rotational elastic energy and explosively accelerate the trunk. Understanding which factors unrelated to the throwing arm are most instrumental in generating high ball velocity also has the potential to help guide training programs to increase pitching performance while potentially protecting the throwing arm from overuse. Therefore, the objective of this study was to compare the spring-like properties of pelvis and trunk rotation during baseball pitching in high school pitchers with varying ball velocities. It was hypothesized that higher-velocity pitchers would be more effective at accelerating the trunk on the pelvis from foot contact to ball release (i.e., releasing the spring).

## 2. Methods

Thirty-five healthy and injury-free high school pitchers participated in this study (age [mean ± SD]: 16.5 ± 1.1 year, height: 1.83 ± 0.06 m, body mass: 82.8 ± 9.9 kg, pitch velocity: 75.0 ± 5.6 mph, right-handed pitchers = 28, left-handed pitchers = 7) as part of a routine preseason fitness evaluation and injury screening. The age range of the subjects was 15.0 to 18.5 years. This study was conducted in accordance with the Declaration of Helsinki and approved by the Northwell Health Institutional Review Board (Study# 21-0878).

After a self-directed warm up, which included stretching of the upper and lower extremities, light tossing into the net and a gradual increase to maximal-effort pitching, thirty-two retro-reflective markers were placed on the lower extremities, arms, trunk and head. The marker locations were as follows: C7 vertebra, right and left acromia, right and left lateral elbow, right and left medial elbow, right and left ulnar styloid, right and left distal radius, right and left ASIS, right and left greater trochanter, sacrum, right and left lateral femoral condyle, right and left medial femoral condyle, right and left lateral malleolus, right and left medial malleolus, right and left base of the 5th metatarsal, and head of the third metacarpal on the throwing hand. Four additional markers were placed on the pitcher’s baseball cap on the front, top and bilateral sides of the head. Each pitcher then threw five fastball pitches into a net positioned approximately thirty feet from a portable indoor turf pitcher’s mound (ProMounds, Braintree, MA, USA). Ball velocity was measured with a radar gun (Pocket Radar Inc., Santa Rosa, CA, USA).

Kinematic data were recorded with a 10-camera motion capture system at 500 Hz (BTS Bioengineering, Quincy, MA, USA). The motion data from the body markers were then filtered with a fourth-order Butterworth low-pass filter with a cutoff frequency of 12 Hz in order to eliminate any high-frequency noise [10]. The global coordinate system was set up such that positive Y was vertically upward and the X direction was orthogonal to Y and positive in the direction of home plate. The Z direction was the cross-product of Y and X and positive in the direction of third base.

A customized model for each pitcher was built using Visual 3D (C-Motion, Inc., Rockville, MD, USA) to compute the angular rotations and velocities of the pelvis and trunk. Instantaneous angular velocities were computed as the first derivative of the angular rotations with respect to time. Segmental coordinate systems were set up in similar fashion to the global coordinate system. Transverse plane motion was defined by rotations about the *Y*-axis, sagittal plane motion was defined by rotations about the *X*-axis and frontal plane motion was defined by rotations about the *Z*-axis. Transverse plane angular positions (degrees) and velocities (degrees/s) for both the pelvis and trunk were positive when rotating towards home plate. For left-handed pitchers, pelvis and trunk transverse plane angular positions were corrected for discontinuities if necessary and converted to the right-handed reference frame by subtracting 180 degrees from all values. The trunk–pelvis separation angle was calculated as the difference in the angular position of the trunk relative to the pelvis in the transverse plane, with positive values indicating that the trunk was lagging behind the pelvis. Transverse plane pelvis and trunk angular positions and velocities were calculated for the three fastest pitches thrown.

The events of stride foot contact, ball release, peak trunk–pelvis separation, peak pelvis velocity and peak trunk velocity were identified for each selected pitch. Stride foot contact was identified as the minimum vertical position of the glove-side medial malleolus marker between the balance point (maximum vertical position of the glove-side medial knee marker) and the end of the pitch (maximum vertical position of the throwing-side lateral malleolus marker). Ball release was identified as the minimum flexion angle of the throwing elbow between the stride foot contact and the end of the pitch. The average accelerations of the pelvis and trunk were calculated for each segment as the difference in velocity between peak velocity and the velocity at foot contact divided by the time from foot contact to peak velocity. Average pelvis and trunk decelerations were calculated by subtracting each segment’s respective velocity at ball release from the peak velocity and dividing by the time from peak velocity to ball release. Additionally, average trunk-on-pelvis acceleration from foot contact (FC) to ball release (BR) was calculated by subtracting the difference of the trunk and pelvis velocities at foot contact from the difference in velocities at ball release and dividing by the time between foot contact and ball release (Equation (1)).

(1) $$\text{Average Trunk-on-pelvis Acceleration}=[(V_{\mathrm{Trunk},\mathrm{BR}}-V_{\mathrm{Pelvis},\mathrm{BR}})-(V_{\mathrm{Trunk},\mathrm{FC}}-V_{\mathrm{Pelvis},\mathrm{FC}})]/{\mathrm{Time}}_{\mathrm{FC}\;\mathrm{to}\;\mathrm{BR}}$$

Associations between ball velocity, trunk-on-pelvis acceleration, subject demographics and the individual transverse plane angular and velocity metrics for the trunk and pelvis were assessed using Pearson correlations. R values between 0.20 and 0.29 indicated a weak relationship, r values between 0.30 and 0.39 indicated a moderate relationship, and r values between 0.40 and 0.69 indicated a strong relationship. R values above 0.70 were indicative of a very strong relationship [11]. *p* values less than 0.05 indicated significant correlations between ball velocity and each kinematic variable.

As part of a secondary analysis to provide further description and practical comparisons, pitchers were classified as higher- (n = 11), average- (n = 11), or lower-velocity (n = 13) based on being above, within, or below 0.5 SDs of the group mean. Single-factor (velocity group) analysis of variance was used to compare subject demographics, trunk–pelvis separation, pelvis velocity and trunk velocity at each event (peak, FC or BR), as well as pelvis, trunk and average trunk-on-pelvis accelerations, between each group. *p*-values < 0.05 were considered significant and were Bonferroni-corrected for post hoc pairwise group comparisons.

## 3. Results

Trunk-on-pelvis acceleration was moderately correlated with ball velocity (r = 0.368, *p* = 0.03) (Figure 1).

Pelvis deceleration was strongly correlated with ball velocity (r = 0.434, *p* = 0.009) (Figure 2) and trunk-on-pelvis acceleration (r = 0.529, *p* = 0.001).

Other factors that were correlated with ball velocity included age (r = 0.433, *p* = 0.009), height (r = 0.448, *p* = 0.007), peak pelvis velocity (r = 0.507, *p* = 0.002), peak trunk velocity (r = 0.413, *p* = 0.014), pelvis velocity at foot contact (r = 0.401, *p* = 0.017) and pelvis velocity at ball release (r = −0.372, *p* = 0.028).

When the pitchers were classified into subgroups, significant differences in demographics, as well as trunk and pelvis kinematics, were apparent. Higher-velocity pitchers were older (*p* = 0.016) and taller (*p* = 0.040) than lower-velocity pitchers, but body mass did not differ between groups (*p* = 0.497) (Table 1).

Peak pelvis and trunk velocities were higher for higher- versus lower-velocity pitchers (*p* = 0.001 and *p* = 0.008, respectively) but did not differ at BR (*p* = 0.229 and *p* = 0.126, respectively). Trunk–pelvis separation metrics did not differ between pitch velocity groups (Table 2).

Compared to lower-velocity pitchers, pelvis deceleration (from peak velocity to BR) and average trunk-on-pelvis acceleration (from FC to BR) were greater in higher-velocity pitchers (*p* = 0.024 and *p* = 0.013, respectively; Figure 3). There were no group differences in trunk acceleration or deceleration by themselves (Effect of Group, *p* = 0.105 and *p* = 0.336, respectively). Additionally, all 11 high-velocity pitchers were able to accelerate the trunk on the pelvis compared to only 18 of the average and low-velocity pitchers (*p* = 0.031). Sample pelvis and trunk velocity graphs for a high-velocity pitcher (Figure 4) and a low-velocity pitcher (Figure 5) are shown to illustrate trunk-on-pelvis acceleration from foot contact to ball release.

## 4. Discussion

The results of this study indicate that higher-velocity high school pitchers achieve greater average trunk-on-pelvis acceleration from foot contact to ball release compared to lower-velocity pitchers. This was accomplished through greater deceleration of the pelvis, which creates a greater relative difference in acceleration between the trunk and pelvis. While numerous studies have shown that higher trunk velocities lead to higher ball velocity [5,6,7,8,12], the current study highlights how these high trunk velocities are achieved. Effective deceleration of the pelvis is needed to transfer angular momentum between the pelvis and trunk (i.e., releasing the torsional spring) and to maintain the difference in velocity between the pelvis and trunk to the point of ball release.

As stated earlier, the study by Fleisig et al. [10] is the only study to quantify trunk-on-pelvis acceleration during the pitching motion. That study, however, was purely descriptive and focused on trunk injury prevention instead of pitching performance. More recently, Tanaka et al. [13] implied a relationship between trunk and pelvis accelerations and decelerations and ball velocity with their finding that trunk velocity at peak pelvis velocity was negatively correlated with ball velocity. Lower trunk velocity at peak pelvis velocity implies greater acceleration of the trunk to reach peak velocity for a given time interval from peak pelvis velocity to peak trunk velocity. During this same time period (from peak pelvis velocity to peak trunk velocity), the pelvis is decelerating, which causes the segments to uncoil and increases the relative acceleration of the trunk on the pelvis. Taken together, the results of the current study and those of Tanaka et al. highlight the spring-like nature of the pelvis-trunk interaction during the pitching motion and offer insights into how high segmental velocities are achieved and transferred distally.

Although a significant difference in average trunk-on-pelvis acceleration was demonstrated between the higher-velocity and lower-velocity pitchers, analysis of the trunk–pelvis separation metrics found no differences between groups. These results are consistent with those of Orishimo et al. [7] and Bullock et al. [6], who both found no association between peak trunk–pelvis separation and ball velocity. Collectively, these results seem to indicate that the ability to control the acceleration and deceleration of the trunk and pelvis to achieve large differences in rotational velocities may be more instrumental in attaining high ball velocities than a high angular separation. According to Equation 1, to achieve maximal average trunk-on-pelvis acceleration, the pelvis must be rotating faster than the trunk at foot contact and the trunk must be rotating with a much higher velocity compared to the pelvis at ball release. This can be done by minimizing trunk motion until after foot contact and effectively decelerating the pelvis before ball release. Thus, dissociating the velocities of the pelvis and trunk throughout the pitch cycle seems to be more important to performance than the positional dissociation of these segments. It should be noted that recent studies by Jump et al. [14] and Wada et al. [15] found positive correlations between ball velocity and trunk–pelvis separation. Therefore, more research into this aspect of the trunk–pelvis interaction is needed.

Studies have shown that the external oblique and rectus abdominus muscles play an integral part in accelerating and decelerating the pelvis and trunk during the pitching motion [10,16,17]. Training these muscles for both explosiveness and eccentric performance is essential for generating high ball velocities and injury prevention. Some common trunk strengthening and core exercises (i.e., medicine ball throws, Russian twists and seated trunk rotations with resistance bands) have been shown to replicate the range of motion observed during pitching while strengthening the abdominals [18]. However, they do not replicate the segmental velocities achieved during pitching. Considering the contributions of pelvis deceleration, and average trunk-on-pelvis acceleration, to ball velocity, high-velocity eccentric training may also be beneficial for pitchers. Deceleration exercises, such as high-speed rotational medicine ball catches, may adapt the spring-like properties of the pelvis and trunk rotational sequencing during pitching. Additional research is needed to develop eccentrically-biased trunk rotation exercises that more closely replicate kinematic patterns seen during the pitching motion and to test their effects on pelvis and trunk kinematics during the pitching delivery.

One limitation to the current study that warrants mention is that the subject pool consisted of a small group (n = 35) of high school baseball pitchers. Therefore, these results may not be applicable to pitchers at the youth, collegiate or professional levels. While one might expect this study group to be mostly homogeneous, the age range was 14.9 to 18.4 years old. As the higher-velocity pitchers were older and taller, it seems that being more physically mature may have helped these pitchers generate greater segmental rotation velocities, accelerations and ball velocities. Additionally, the older pitchers may have had more experience training and have refined their technique to achieve this level of performance. More research into how pitchers at different levels of development store and release elastic energy and the effects on performance is needed.

A second limitation was the fact that the spring-like mechanism between the pelvis and trunk was not directly measured or quantified. While spring-like properties can be inferred from the angular separation between these segments and the differences in velocity during the pitching motion, no direct measurements of neuromuscular function or rotational kinetics were made. Future research is needed to measure stretch-shortening cycle function during the pitching motion and to directly quantify the spring-like properties generated between the segments.

The current study highlights not only the importance of achieving high pelvis and trunk velocities in order to generate high ball velocities but also controlling the speeds of these segments such that the optimal trunk-on-pelvis acceleration is reached. Higher-velocity pitchers achieved greater average trunk-on-pelvis acceleration by more effectively decelerating the pelvis. This likely aided in the transfer of momentum between the pelvis and trunk (i.e., releasing the torsional spring) and helped to maintain the difference in velocity between the pelvis and trunk to the point of ball release. Therefore, training the abdominal musculature to more effectively control the speeds of the trunk and pelvis throughout the pitching motion may lead to an increase in ball velocity without overusing the throwing arm.

## Figures and Tables

**Figure 1:**
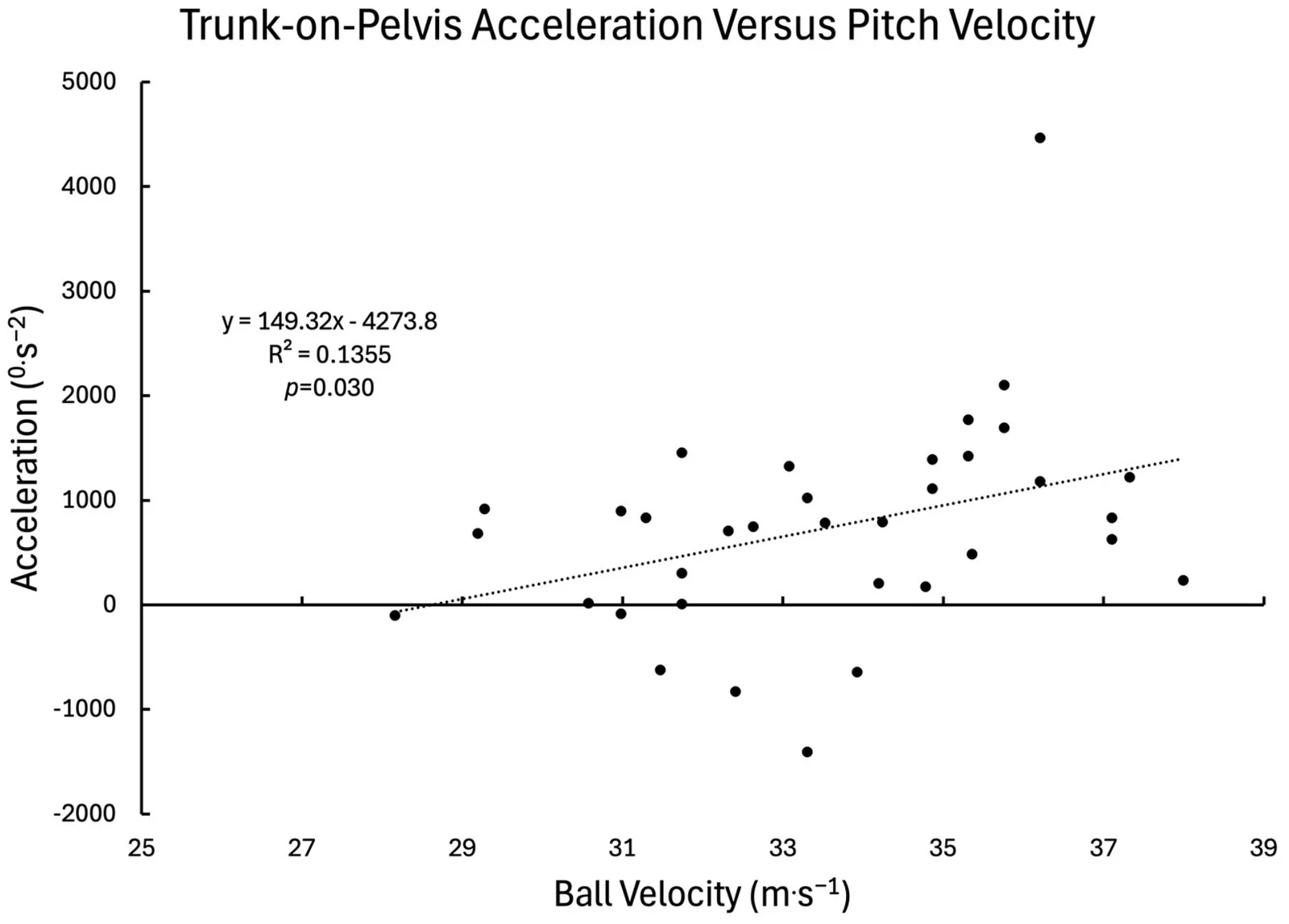
The relationship between trunk-on-pelvis acceleration and ball velocity.

**Figure 2:**
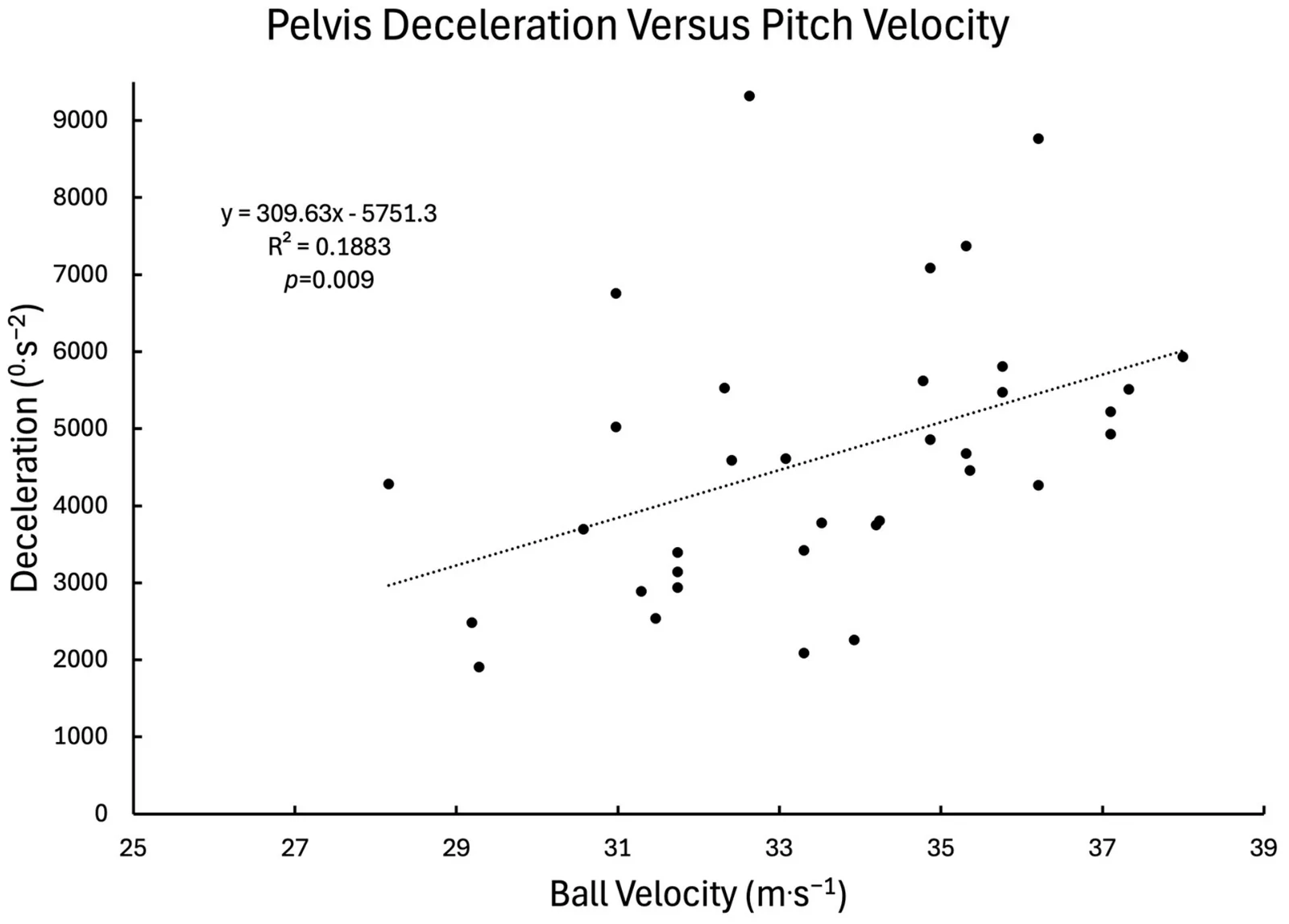
The relationship between pelvis deceleration and ball velocity.

**Figure 3:**
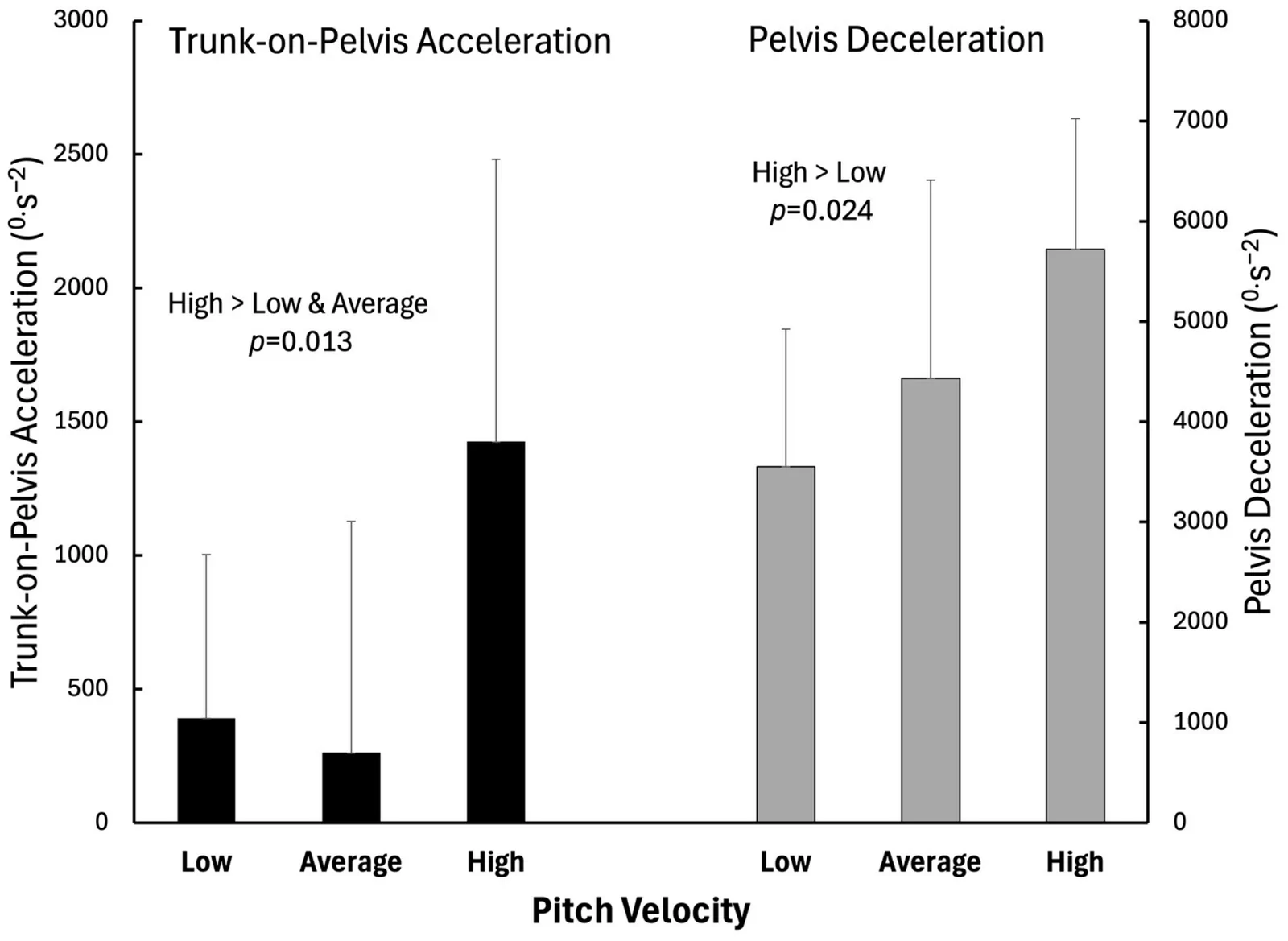
Comparison of average trunk-on-pelvis acceleration and pelvis deceleration between groups. Average trunk-on-pelvis acceleration from FC to BR (Effect of Pitch Group *p* = 0.011); pelvis deceleration from peak pelvis velocity to BR (Effect of Pitch Group *p* = 0.029).

**Figure 4:**
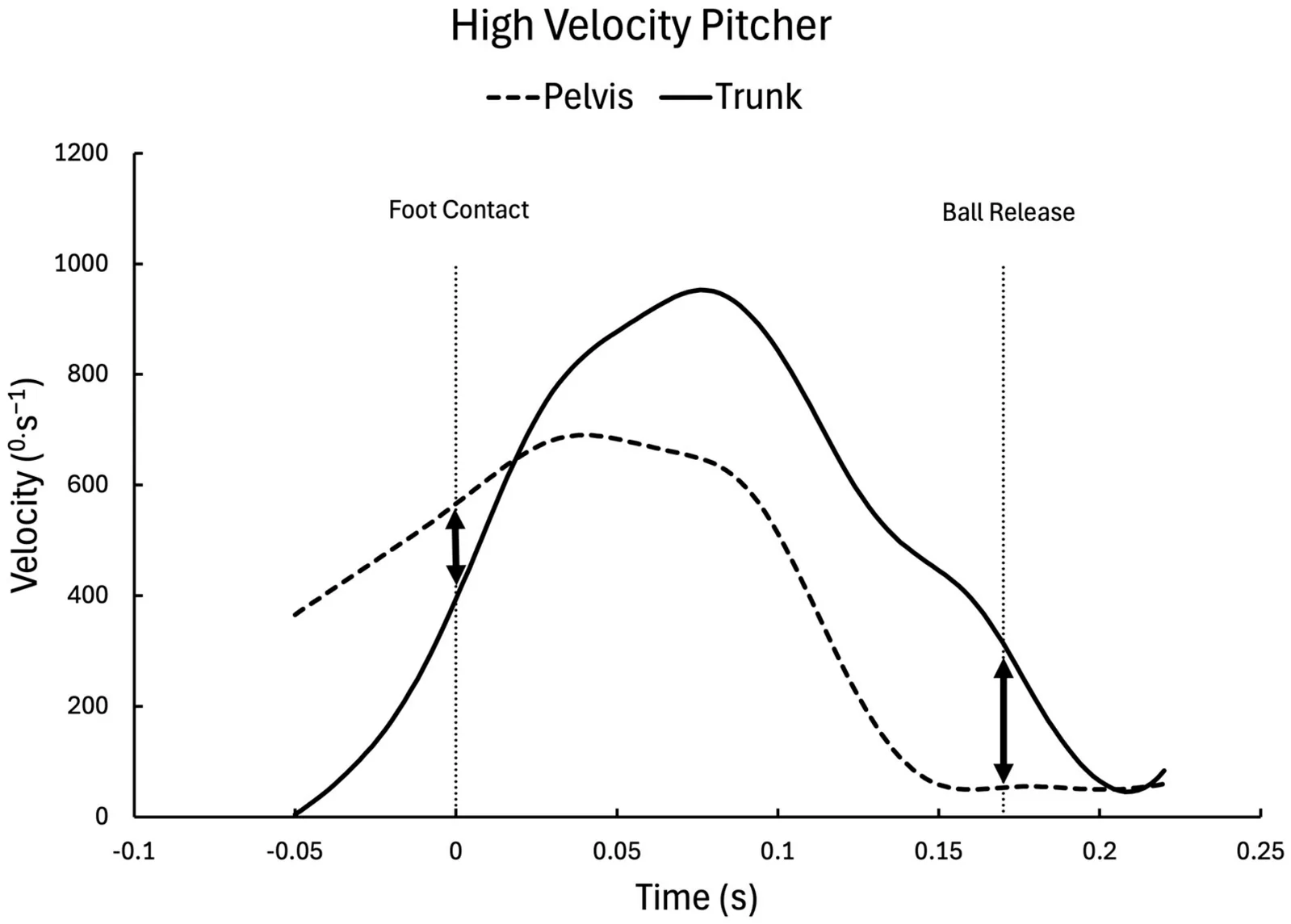
Pelvis and trunk velocities of a representative high-velocity pitcher from foot contact to ball release. The vertical arrows highlight the difference in trunk and pelvis velocities at foot contact and ball release. At foot contact the pelvis is rotating faster than the trunk. At ball release the trunk is rotating faster than the pelvis. Therefore, from foot contact to ball release the trunk is accelerating on the pelvis.

**Figure 5:**
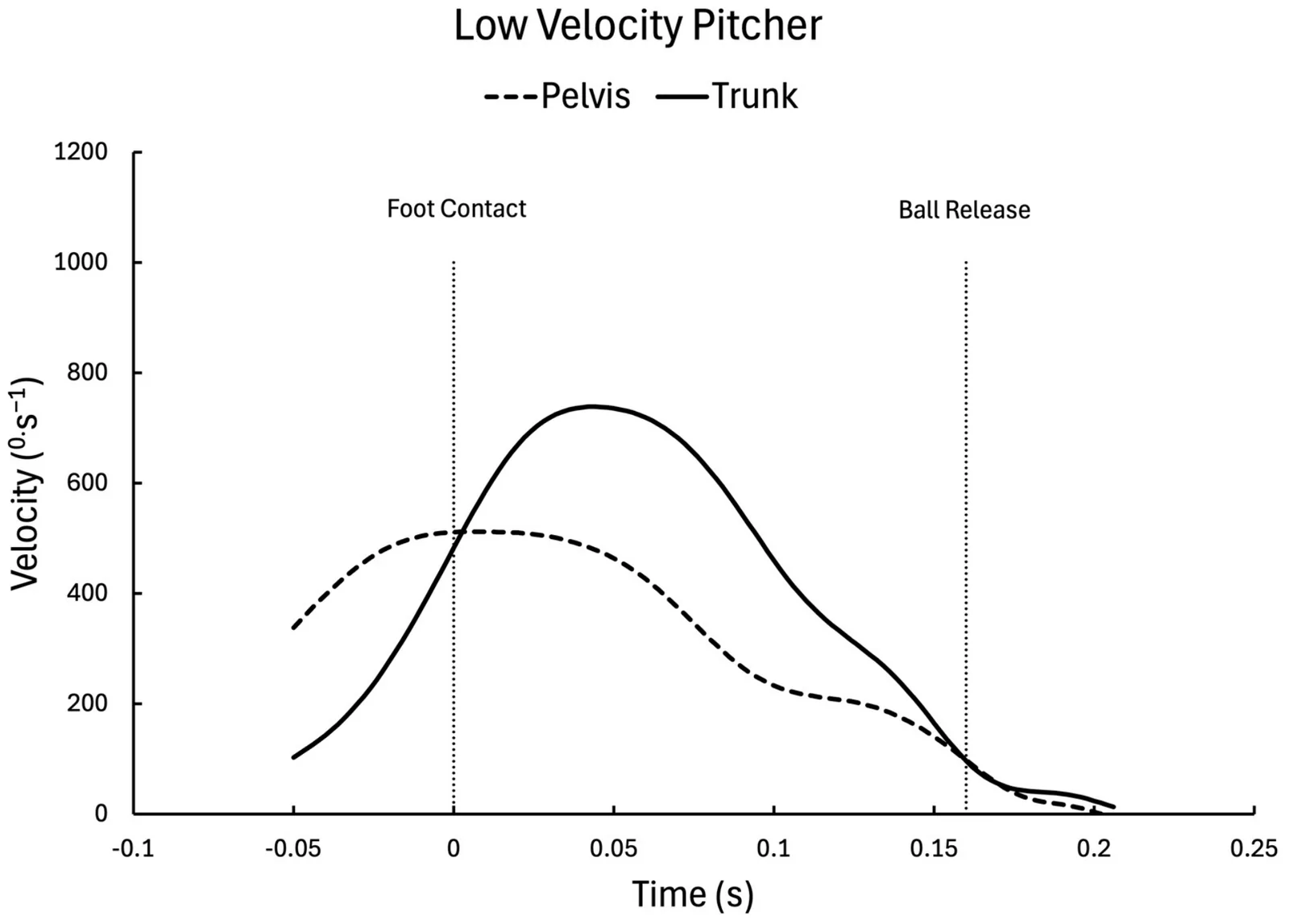
Pelvis and trunk velocities of a representative low-velocity pitcher from foot contact to ball release. At foot contact there is minimal difference in pelvis and trunk velocities. At ball release there is also minimal difference between pelvis and trunk velocities. Therefore, from foot contact to ball release the trunk is not accelerating on the pelvis.

**Table 1:** Group demographics of higher-, average- and lower-velocity pitchers.

Group	Age (Years)	Height (m)	Body Mass (kg)	Ball Velocity (m·s^−1^)
Low	16.0 ± 1.2	1.79 ± 0.07	82.7 ± 14.0	30.9 ± 1.3
Average	16.7 ± 0.7	1.83 ± 0.07	80.5 ± 5.2	33.9 ± 0.8 ^†^
High	17.2 ± 0.8 *	1.87 ± 0.03 *	86.0 ± 7.7	36.3 ± 0.9 *^,††^
*GROUP Effect*	**0.016**	**0.040**	0.497	**<0.001**

ANOVA *p*-value is for the Main Effect of Group. * Higher-velocity group is significantly greater than lower-velocity group (*p* < 0.05). ^†^ Average-velocity group is significantly greater than lower-velocity group (*p* < 0.05). ^††^ Higher-velocity group is significantly greater than average-velocity group (*p* < 0.05).

**Table 2:** Comparisons of trunk–pelvis separation, pelvis velocity and trunk velocity among higher-, average- and lower-velocity pitchers.

	Trunk–Pelvis Separation (°)			Pelvis Velocity (°·s^−1^)			Trunk Velocity (°·s^−1^)		
Group	Foot Contact	Peak	Ball Release	Foot Contact	Peak	Ball Release	Foot Contact	Peak	Ball Release
Low	26 ± 10	26 ± 10	−4 ± 7	518 ± 76	547 ± 64	135 ± 94	596 ± 123	712 ± 83	259 ± 137
Average	21 ± 6	21 ± 8	−2 ± 5	585 ± 98	632 ± 122	107 ± 72	617 ± 85	747 ± 111	215 ± 50
High	33 ± 17	34 ± 18	−4 ± 7	615 ± 64 *	694 ± 75 *	79 ± 62	640 ± 110	849 ± 113 *	312 ± 111
*GROUP Effect*	0.058	0.076	0.802	**0.018**	**0.001**	0.229	0.609	**0.008**	0.126

ANOVA *p*-value is for the Main Effect of Group. * Higher-velocity group significantly greater than lower-velocity group (*p* < 0.05).

## Data Availability

The raw data supporting the conclusions of this article will be made available by the authors on request.
